# National Trends in the Prevalence of Unmet Health Care and Dental Care Needs During the COVID-19 Pandemic: Longitudinal Study in South Korea, 2009-2022

**DOI:** 10.2196/51481

**Published:** 2024-09-18

**Authors:** Yeji Kim, Soeun Kim, Somin Lee, Jaeyu Park, Ai Koyanagi, Lee Smith, Min Seo Kim, Guillaume Fond, Laurent Boyer, Guillermo Felipe López Sánchez, Elena Dragioti, Hyeon Jin Kim, Hayeon Lee, Yejun Son, Minji Kim, Sunyoung Kim, Dong Keon Yon

**Affiliations:** 1 Department of Medicine Kyung Hee University College of Medicine Seoul Republic of Korea; 2 Center for Digital Health Medical Science Research Institute Kyung Hee University College of Medicine Seoul Republic of Korea; 3 Department of Precision Medicine Kyung Hee University College of Medicine Seoul Republic of Korea; 4 Department of Regulatory Science Kyung Hee University Seoul Republic of Korea; 5 Research and Development Unit Parc Sanitari Sant Joan de Deu Barcelona Spain; 6 Centre for Health, Performance and Wellbeing Anglia Ruskin University Cambridge United Kingdom; 7 Medical and Population Genetics and Cardiovascular Disease Initiative Broad Institute of MIT and Harvard Cambridge, MA United States; 8 Research Centre on Health Services and Quality of Life Assistance Publique-Hôpitaux de Marseille Aix Marseille University Marseille France; 9 Division of Preventive Medicine and Public Health, Department of Public Health Sciences School of Medicine University of Murcia Murcia Spain; 10 Research Laboratory Psychology of Patients, Families, and Health Professionals Department of Nursing, School of Health Sciences University of Ioannina Ioannina Greece; 11 Department of Family Medicine Kyung Hee University Medical Center Kyung Hee University College of Medicine Seoul Republic of Korea; 12 Department of Pediatrics Kyung Hee University Medical Center Kyung Hee University College of Medicine Seoul Republic of Korea

**Keywords:** COVID-19, pandemic, epidemiology, South Korea, unmet health care, unmet dental care.

## Abstract

**Background:**

Although previous studies have investigated trends in unmet health care and dental care needs, most have focused on specific groups, such as patients with chronic conditions and older adults, and have been limited by smaller data sets.

**Objective:**

This study aims to investigate the trends and relative risk factors for unmet health care and dental care needs, as well as the impact of the COVID-19 pandemic on these needs.

**Methods:**

We assessed unmet health care and dental care needs from 2009 to 2022 using data from the Korea Community Health Survey (KCHS). Our analysis included responses from 2,750,212 individuals. Unmet health care or dental care needs were defined as instances of not receiving medical or dental services deemed necessary by experts or desired by patients.

**Results:**

From 2009 to 2022, the study included 2,700,705 individuals (1,229,671 men, 45.53%; 673,780, 24.95%, aged 19-39 years). Unmet health care needs decreased before the COVID-19 pandemic; however, during the pandemic, there was a noticeable increase (β_diff_ 0.10, 95% CI 0.09-0.11). Unmet dental care needs declined before the pandemic and continued to decrease during the pandemic (β_diff_ 0.23, 95% CI 0.22-0.24). Overall, the prevalence of unmet dental care needs was significantly higher than that for unmet health care needs. While the prevalence of unmet health care needs generally decreased over time, the β difference during the pandemic increased compared with prepandemic values.

**Conclusions:**

Our study is the first to analyze national unmet health care and dental care needs in South Korea using nationally representative, long-term, and large-scale data from the KCHS. We found that while unmet health care needs decreased during COVID-19, the decline was slower compared with previous periods. This suggests a need for more targeted interventions to prevent unmet health care and dental care needs.

## Introduction

Unmet health care and dental care needs significantly impact citizens’ quality of life and welfare. The SARS-CoV-2, which affects the respiratory system, has dramatically influenced the use of medical services, resulting in decreased hospital visits and hospitalization rates in many countries [1-3]. Experts attribute this to factors such as anxiety, concerns about infection in hospitals, and executive orders to close hospitals, rather than a decrease in the actual number of patients [1,4,5].

Unmet medical and oral care can exacerbate health conditions, making timely access to medical services crucial. Patients with chronic diseases, who require continuous care, face significant health risks when their medical needs are not met. Therefore, during the COVID-19 pandemic, it is important to identify trends and vulnerable groups experiencing delayed hospital visits despite needing treatment. Preliminary analysis suggests that socioeconomic status, geographical location, and preexisting health conditions may be significant risk factors for unmet health care and dental care needs, highlighting the complexity of the issue [6,7].

Several studies have examined unmet health care needs, but many are short-term investigations or involve small sample sizes, focusing on groups such as older adults, the disabled, and individuals with specific diseases [8-10]. Similarly, research on unmet dental needs has often been conducted over a relatively short period or within a specific group [11,12]. Additionally, while experts have noted the decrease in hospital use after COVID-19, no studies have been found that compare the periods before and after the pandemic or analyze the associated risk factors [4,13]. Moreover, only a few studies have investigated unmet health care and dental care needs. Therefore, it was necessary to examine the long-term trends of unmet health care and dental care needs and identify related risk factors for the entire population of Korea. Understanding vulnerable groups in the context of rapidly changing medical policies and restrictions after COVID-19 is essential for future predictions.

Recent studies have highlighted various aspects of this issue. For example, research has discussed the heightened barriers to accessing health care services during the pandemic, particularly for low-income families [14]. Additionally, unmet medical needs during the pandemic have significantly impacted mental health, with many individuals experiencing increased anxiety and depression due to delayed or foregone medical care [15]. Furthermore, disruptions in dental care have been significant, with many patients unable to receive necessary treatments, underscoring the need for targeted interventions to address these gaps [16].

Thus, our study aims to confirm the trends, relative risk factors, and impact of the COVID-19 pandemic on unmet health care and dental care needs using nationally representative data from the Korea Community Health Survey (KCHS) for the years 2009 to 2022 [17].

## Methods

### Data

**Figure 1:**
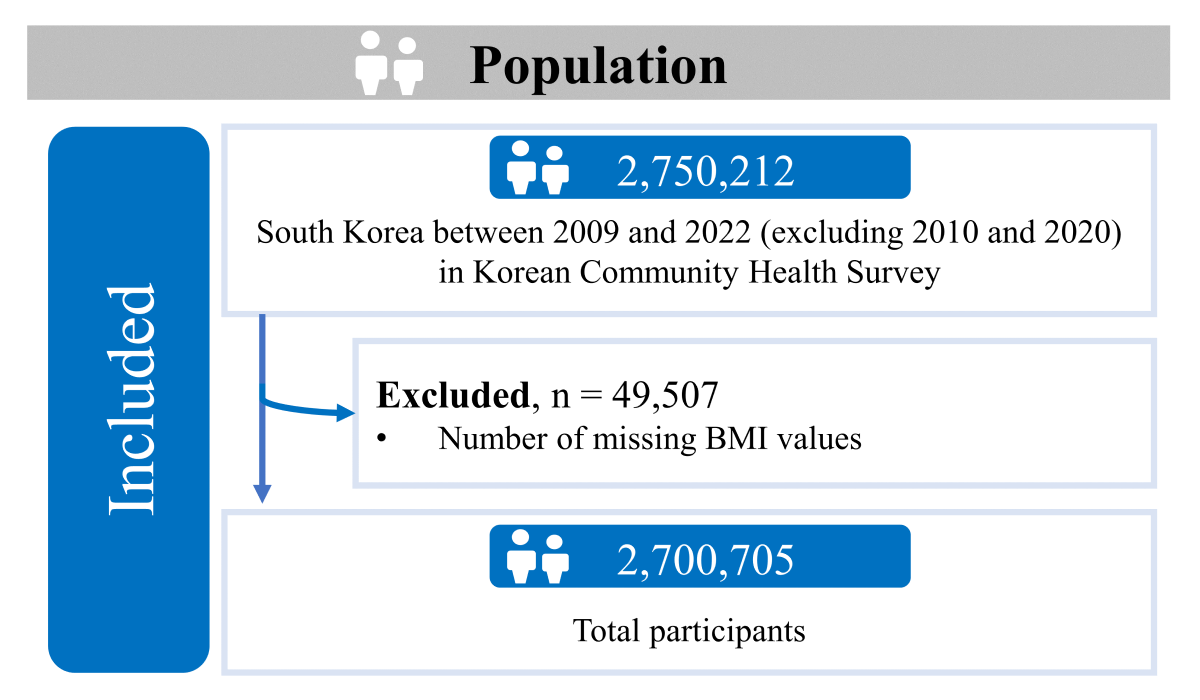
Study participants within the total population.

This study used data from the KCHS for the years 2009 and 2011 through 2019, and 2021 through 2022 (excluding 2010 and 2020) [18,19]. The KCHS is an annual initiative conducted by trained interviewers through household visits. This self-reporting survey targets approximately 900 adults aged 19 years and older at each of the 255 public health centers nationwide. It covers 18 domains and includes 163 health-related questions. In 2022, a comprehensive survey was conducted involving a total of 227,279 individuals. Out of the 163 survey questions, 18 focused on unmet health care and dental care needs. The analysis included factors such as age, sex, marital status, education, occupation, income status, residence, primary livelihood security recipient, smoking status, alcohol consumption, weekly walks, self-rated health, depression, BMI, and previous diagnoses of diabetes mellitus and hypertension. Data on unmet dental care needs for 2010 and 2020 were unavailable for this study. In total, 2,700,705 participants were included, with 1,229,671 (45.5%) being men (Figure 1).

### Ethics Approval

The KCHS data were anonymized, and the study protocol received approval from the Institutional Review Board of the Korea Disease Control and Prevention Agency. All participants provided informed consent, and the study was conducted in accordance with the principles of the Declaration of Helsinki (approval numbers 2010-02CON-22-P, 2011-05CON04-C, 2012-07CON-01-2C, 2013-06EXP-01-3C, 2014-08EXP-09-​4CA, and 2016-10-01-TA).

### Analytic Framework

The Andersen Health Care Utilization Model is a well-established theoretical framework that systematically analyzes both social and personal factors to explain determinants of health service utilization [20]. According to the model, 3 dynamics—predisposing, enabling, and need variances—determine the consumption of health services, including outpatient and inpatient treatment. Predisposing factors are sociodemographic traits that increase an individual’s demand for health care, such as age, ethnicity, sex, and socioeconomic status. For example, individuals are more likely to seek care if they believe that using health services effectively addresses their illness. Enabling factors encompass community support, availability of health insurance, and family support. The demand for care reflects both one’s actual and perceived need for medical services. We referenced the Andersen Health Care Utilization Model to identify inequalities in access to health services and to determine how various factors contribute to the utilization of these services.

### Dependent Variable

Unmet health care and dental care needs were defined as instances where individuals did not receive the medical or dental services deemed necessary by experts or desired by the patients [4]. Subjective unmet health was analyzed based on the question “Have you ever needed health care (test or treatment) in this year but not received medical treatment?” and subjective unmet dental care was analyzed based on the question “Have you ever needed dental care (test or treatment) in this year but not received medical treatment?” [21].

### Independent Variable

The survey period was treated as the independent variable and divided into 5 time segments to ensure estimation stability: 2009-2011 (excluding 2010), 2012-2014, 2015-2017, 2018-2019, and 2021-2022. The years 2021 and 2022 were designated as the COVID-19 pandemic period.

### Covariates

The following covariates were considered as predisposing factors: age groups (19-39, 40-59, and ≥60 years), sex, marital status, education level (elementary school or lower, middle school, high school, and college or higher), residence (urban and rural) [22], and occupation (unemployed, blue-collar, and white-collar). Enabling/disabling factors included income status (unknown, <3 million KRW, 3-5 million KRW, and ≥5 million KRW per month; 1 KRW=US $0.00073) and being a basic livelihood security recipient. Need factors included smoking status, alcohol consumption (none, monthly, and weekly), weekly physical activity (rarely, 1-2, 3-4, and ≥5 times per week), subjective health status (bad, normal, and good), depression, previous diagnoses of diabetes mellitus or hypertension (yes or no), and BMI categories (underweight [<18.5 kg/m^2^], normal [18.5-22.9 kg/m^2^], overweight [23.0-24.9 kg/m^2^], and obese [≥25.0 kg/m^2^]) [23-26].

### Statistical Analyses

We investigated overall population characteristics and calculated the weighted prevalence of unmet health care and dental care needs for each subgroup. To obtain national prevalence estimates, we performed a comprehensive sample analysis, accounting for stratification, clustering, and weighting. The methodology for calculating weights was carefully designed to reflect the complex survey design of the KCHS [27]. Weights assigned to each respondent were used to adjust for differences in selection probabilities and to align with the age and sex distribution of the Korean population [27]. The weighting process involved several steps, including the calculation of design weights, poststratification weights, and final weights [27]. A weighted linear regression model was used to determine the trend of unmet health care and dental care needs in the prepandemic and pandemic periods, identifying differences in coefficients between these periods. The prevalence and trends of unmet health care and dental care needs were analyzed accordingly. Finally, prevalence ratios (PRs) were computed to evaluate the interaction term for each risk factor, separately for the prepandemic and pandemic periods [28-30]. This analytical approach allows for interpreting which demographic or risk groups exhibited heightened vulnerability to unmet health care or dental care needs during both the prepandemic and pandemic periods. All statistics are presented as weighted percentages with 95% CIs [31]. Statistical significance was defined as a 2-sided *P* value of <.05 [32-35]. Statistical analyses were performed using SAS (version 9.4; SAS Institute).

## Results

After excluding outliers, we analyzed data from 2,700,705 respondents with complete survey responses. The sample was distributed across different survey periods, as shown in Table 1: 455,909 participants from 2009 to 2011 (excluding 2010); 679,865 participants from 2012 to 2014; 678,560 participants from 2015 to 2017; 435,091 participants from 2018 to 2019; 224,001 participants in 2021; and 227,279 participants in 2022. Table 1 summarizes the weighted distributions of each dependent variable for participants. Complex sample weights were used to obtain more accurate population estimates.

**Figure 2:**
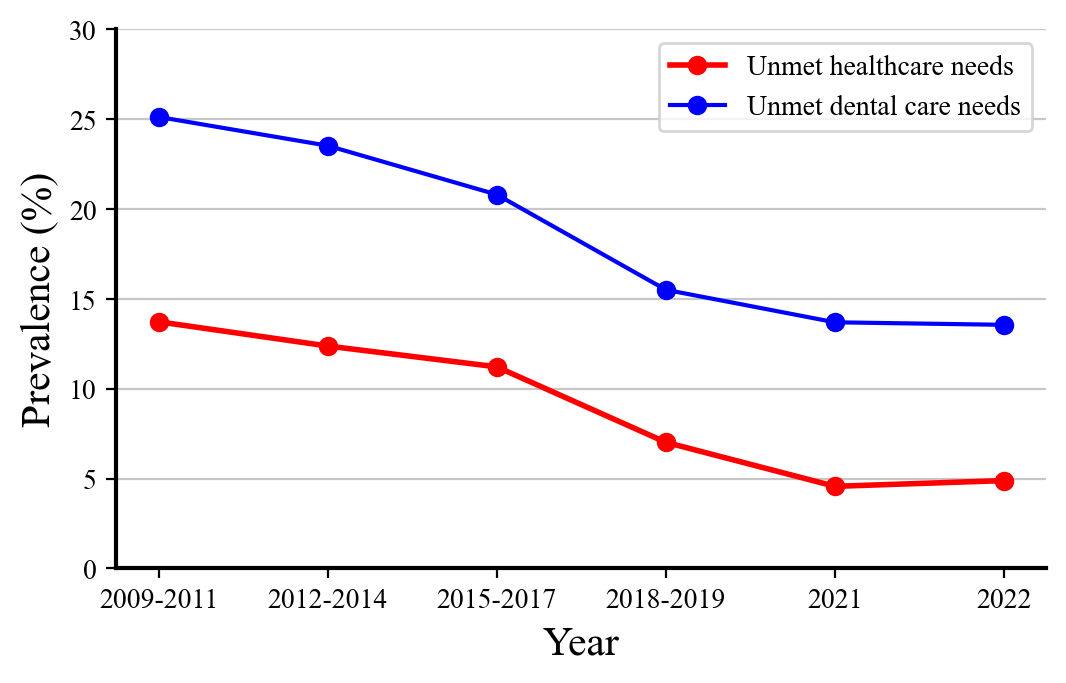
Trends in the overall prevalence of unmet health care and dental care needs among Korean adults, 2009-2022.

Table 2 and Figure 2 show the weighted prevalence and 95% CIs of unmet health care needs for each demographic characteristic, along with the trends before and during the pandemic. The β difference from the linear regression model, comparing the prepandemic and pandemic periods, along with its 95% CI, indicates the impact of COVID-19 on these trends. Overall, the prevalence of unmet health care needs decreased from 14.06% (64,108/455,909) in 2009-2011 to 4.82% (10,790/224,001) in 2021, with a slight increase to 5.28% (11,990/227,279) in 2022. While most subgroups exhibited similar patterns, the unemployed subgroup within the occupation category continued to experience a decline (β_diff_ 0.15, 95% CI 0.14-0.16) during the same period.

Table 3 and Figure 2 display the weighted prevalence and 95% CIs of unmet dental care needs. We observed a higher prevalence of unmet dental care needs compared with health care needs. Although the failure rate to meet dental care needs generally declined from 24.46% (111,526/455,909) in 2009-2011 to 14.02% (31,864/227,279) in 2022, the β difference during the pandemic increased compared with prepandemic values (β_diff_ 0.23, 95% CI 0.22-0.24).

Table 4 presents the risk factors for unmet health care needs before and during the pandemic. Women experienced unmet health care needs more frequently than men both before (PR 1.04, 95% CI 1.03-1.04) and during the pandemic (PR 1.02, 95% CI 1.01-1.02). Similarly, basic livelihood security recipients were 1.13 times more likely to experience unmet health care needs before the pandemic (95% CI 1.12-1.15) and 1.05 times more likely during the pandemic (95% CI 1.04-1.07) compared with nonrecipients.

Lastly, Table 5 presents the risk factors for unmet dental care needs before and during the pandemic. Women experienced unmet dental care needs more frequently than men both before (PR 1.04, 95% CI 1.03 to 1.04) and during the pandemic (PR 1.02, 95% CI 1.01-1.03). Similarly, basic livelihood security recipients had a higher PR for unmet dental care needs compared with nonrecipients, with a PR of 1.24 before the pandemic (95% CI 1.21-1.26) and 1.16 during the pandemic (95% CI 1.13-1.19).

**Table 1:** Overall characteristics of the participants (N=2,700,705).

Characteristics		Before the pandemic					During the pandemic	
		2009-2011	2012-2014	2015-2017	2018-2019	2021		2022
Overall, n (%)		455,909 (16.88)	679,865 (25.17)	678,560 (25.13)	435,091 (16.11)	224,001 (8.29)		227,279 (8.4)
**Age, years, n (%)**								
	19-39	134,775 (29.56)	178,580 (26.27)	165,271 (24.31)	98,942 (22.74)	49,510 (22.10)		46,702 (20.55)
	40-59	178,499 (39.15)	267,116 (39.29)	256,528 (37.73)	158,399 (36.41)	77,892 (34.77)		77,065 (33.91)
	≥60	142,635 (31.29)	234,169 (34.44)	256,761 (37.77)	177,750 (40.85)	96,599 (43.12)		103,512 (45.54)
**Sex, n (%)**								
	Men	208,930 (45.83)	307,312 (45.20)	306,356 (45.06)	199,171 (45.78)	103,111 (46.03)		104,791 (46.11)
	Women	246,979 (54.17)	372,553 (54.80)	372,204 (54.75)	235,920 (54.22)	120,890 (53.97)		122,488 (53.89)
**Marriage, n (%)**								
	Married	322,859 (79.82)	480,169 (79.63)	468,633 (69.06)	300,449 (69.05)	149,282 (66.64)		150,928 (66.41)
	Unmarried	133,050 (29.18)	199,696 (29.37)	209,927 (30.94)	134,642 (30.95)	74,719 (33.36)		76,351 (33.59)
**Region of residence, n (%)**								
	Urban	217,549 (47.72)	326,980 (48.09)	325,575 (47.89)	213,956 (49.18)	109,047 (48.68)		110,885 (48.79)
	Rural	238,360 (52.28)	352,885 (51.91)	352,985 (51.92)	221,135 (50.82)	114,954 (51.32)		116,394 (51.21)
**Household income, n (%)**								
	Unknown	25,350 (5.56)	23,123 (3.40)	6375 (0.94)	77,739 (17.87)	47,729 (21.31)		45,027 (19.81)
	Low (<3 million KRW^a^ per month)	258,676 (56.74)	361,581 (53.18)	371,658 (54.67)	164,760 (37.87)	82,715 (36.93)		80,927 (35.61)
	Middle (3-5 million KRW per month)	110,734 (24.29)	175,003 (25.74)	183,909 (27.05)	93,788 (21.56)	42,891 (19.15)		43,619 (19.19)
	High (≥5 million KRW per month)	61,149 (13.41)	120,158 (17.67)	116,618 (17.15)	98,804 (22.71)	50,666 (22.62)		57,706 (25.39)
**Basic livelihood security recipient, n (%)**								
	Yes	17,928 (3.93)	22,299 (3.28)	22,546 (3.32)	14,035 (3.22)	9119 (4.07)		9953 (4.38)
	No	437,981 (96.07)	657,566 (96.72)	656,014 (96.49)	421,056 (96.77)	214,882 (95.93)		217,326 (95.62)
**Occupation, n (%)**								
	White-collar	84,042 (18.43)	129,959 (19.12)	132,380 (19.47)	85,043 (19.55)	46,668 (20.83)		47,592 (22.94)
	Blue-collar	195,810 (42.95)	297,924 (43.82)	298,092 (43.85)	187,701 (43.14)	94,271 (42.09)		95,792 (42.15)
	Unemployed	176,057 (38.62)	251,982 (37.06)	248,088 (36.49)	162,347 (37.31)	83,062 (37.08)		83,895 (36.91)
**Education, n (%)**								
	Elementary school or lower	124,134 (27.23)	177,052 (26.04)	166,751 (24.53)	96,557 (22.19)	44,964 (20.07)		45,342 (19.95)
	Middle school	54,204 (11.89)	77,586 (11.41)	76,518 (11.25)	50,426 (11.59)	24,518 (10.95)		25,936 (11.41)
	High school	135,043 (29.62)	195,506 (28.76)	192,153 (28.26)	127,274 (29.25)	65,685 (29.32)		66,728 (29.36)
	College or higher	142,528 (31.26)	229,721 (33.79)	243,138 (35.76)	160,834 (36.97)	88,834 (39.66)		89,273 (39.28)
**Smoking status, n (%)**								
	Nonsmoking	353,134 (77.46)	540,609 (79.52)	555,763 (81.75)	359,805 (82.70)	187,607 (83.75)		189,814 (83.52)
	Smoking	102,775 (22.54)	139,256 (20.48)	122,797 (18.06)	75,286 (17.30)	36,394 (16.25)		37,465 (16.48)
**Alcohol** **consumption** **frequency, n (%)**								
	No	223,919 (49.11)	329,241 (48.43)	324,498 (47.73)	212,834 (48.92)	109,234 (48.76)		100,333 (44.15)
	Monthly	136,669 (29.98)	204,156 (30.03)	204,420 (30.07)	127,722 (29.36)	49,165 (21.95)		56,089 (24.68)
	Weekly	95,321 (20.91)	146,468 (21.54)	149,642 (22.01)	94,535 (21.73)	65,602 (29.29)		70,857 (31.18)
**Walking per week, n (%)**								
	Rarely	98,560 (21.62)	164,556 (24.20)	160,684 (23.63)	87,565 (20.13)	45,308 (20.23)		42,403 (18.66)
	1-2 times	44,517 (9.76)	81,093 (11.93)	80,638 (11.86)	54,038 (12.42)	28,923 (12.91)		25,049 (11.02)
	3-4 times	63,994 (14.04)	104,281 (15.34)	106,400 (15.65)	70,409 (16.18)	39,788 (17.76)		38,098 (16.76)
	≥5 times	248,838 (54.58)	329,935 (48.53)	330,838 (48.66)	223,079 (51.27)	109,982 (49.10)		121,729 (53.56)
**Subjective health status, n (%)**								
	Good	186,523 (40.91)	258,536 (38.03)	249,927 (36.76)	153,545 (35.29)	91,952 (41.05)		92,270 (40.60)
	Normal	174,616 (38.30)	276,698 (40.70)	284,894 (41.90)	195,805 (45.00)	95,389 (42.58)		93,891 (41.31)
	Bad	94,770 (20.79)	144,631 (21.27)	143,739 (21.14)	85,741 (19.71)	36,660 (16.37)		41,118 (18.09)
**Depression, n (%)**								
	No	427,015 (93.66)	639,125 (94.01)	635,929 (93.54)	409,513 (94.12)	208,048 (92.88)		210,121 (92.45)
	Yes	28,894 (6.34)	40,740 (5.99)	42,631 (6.27)	25,578 (5.88)	15,953 (7.12)		17,158 (7.55)
**Hypertension, n (%)**								
	No	96,583 (21.18)	163,227 (24.01)	178,829 (26.30)	119,828 (27.54)	63,601 (28.39)		68,255 (30.03)
	Yes	359,326 (78.82)	516,638 (75.99)	499,731 (73.50)	315,263 (72.46)	160,400 (71.61)		159,024 (69.97)
**Diabetes mellitus, n (%)**								
	No	35,546 (7.80)	62,111 (9.14)	71,190 (10.47)	48,132 (11.06)	27,405 (12.23)		30,028 (13.21)
	Yes	420,363 (92.20)	617,754 (90.86)	607,370 (89.34)	386,959 (88.94)	196,596 (87.77)		197,251 (86.79)
**BMI group, n (%)**								
	Underweight (<18.5 kg/m^2^)	41,352 (9.07)	68,482 (10.07)	57,814 (8.50)	25,388 (5.84)	9860 (4.40)		10,224 (4.50)
	Normal (18.5-22.9 kg/m^2^)	203,232 (44.58)	289,865 (42.64)	278,254 (40.93)	161,248 (37.06)	89,928 (40.15)		90,633 (39.88)
	Overweight (23.0-24.9 kg/m^2^)	104,848 (23.00)	155,858 (22.92)	159,679 (23.49)	104,424 (24.00)	54,523 (24.34)		55,235 (24.30)
	Obese (≥25.0 kg/m^2^)	106,477 (23.35)	165,660 (24.37)	182,813 (26.89)	144,031 (33.10)	69,690 (31.11)		71,187 (31.32)

^a^1 KRW=US $0.00073.

**Table 2:** Prevalence and trends for unmet health care needs.^a^

Characteristics		Total, weighted percentage (95% CI)	Before the pandemic, weighted percentage (95% CI)					During the pandemic, weighted percentage (95% CI)			Trends before the pandemic, β-coefficient (95% CI)^b^		Trends during the pandemic, β-coefficient (95% CI)^b^		Difference in trends, β-coefficient (95% CI)^b^
			2009-2011	2012-2014	2015-2017	2018-2019	2021		2022						
Overall		10.33 (10.27 to 10.39)	13.73 (13.55 to 13.90)	12.38 (12.26 to 12.51)	11.22 (11.10 to 11.34)	7.02 (6.89 to 7.15)	4.57 (4.43 to 4.70)		4.89 (4.75 to 5.03)	–*0.21 (–0.22 to –0.21)*		–*0.11 (–0.12 to –0.11)*		*0.10 (0.90 to 0.11)*	
**Age (years)**															
	19-39	11.48 (11.38 to 11.59)	13.71 (13.46 to 13.96)	13.55 (13.34 to 13.76)	13.11 (12.90 to 13.32)	7.85 (7.63 to 8.08)	4.38 (4.15 to 4.62)		4.88 (4.63 to 5.12)	–*0.15 (–0.16 to –0.14)*		–*0.18 (–0.19 to –0.17)*		–*0.30 (–0.40 to –0.20)*	
	40-59	10.39 (10.30 to 10.47)	13.66 (13.42 to 13.90)	12.17 (11.99 to 12.34)	11.13 (10.96 to 11.30)	7.24 (7.05 to 7.43)	5.03 (4.81 to 5.25)		5.35 (5.13 to 5.58)	–*0.19 (–0.19 to –0.18)*		–*0.11 (–0.12 to –0.10)*		*0.80 (0.70 to 0.90)*	
	≥60	8.48 (8.39 to 8.57)	13.90 (13.60 to 14.20)	10.74 (10.54 to 10.94)	8.54 (8.37 to 8.72)	5.57 (5.40 to 5.74)	4.15 (3.96 to 4.35)		4.31 (4.13 to 4.50)	–*0.28 (–0.29 to –0.28)*		–*0.60 (–0.70 to –0.50)*		*0.22 (0.21 to 0.23)*	
**Sex**															
	Men	8.88 (8.80 to 8.96)	11.87 (11.67 to 12.08)	10.54 (10.38 to 10.70)	9.69 (9.54 to 9.85)	6.09 (5.93 to 6.25)	3.79 (3.62 to 3.95)		4.22 (4.05 to 4.40)	–*0.18 (–0.18 to –0.17)*		–*0.09 (–0.10 to –0.08)*		*0.90 (0.08 to 0.10)*	
	Women	11.77 (11.69 to 11.85)	15.55 (15.33 to 15.77)	14.21 (14.04 to 14.38)	12.73 (12.57 to 12.89)	7.95 (7.78 to 8.12)	5.33 (5.14 to 5.53)		5.55 (5.36 to 5.74)	–*0.25 (–0.25 to –0.24)*		–*0.12 (–0.12 to –0.11)*		*0.13 (0.11 to 0.15)*	
**Marriage**															
	Married	10.01 (9.94 to 10.08)	13.41 (13.22 to 13.60)	11.93 (11.79 to 12.08)	10.70 (10.56 to 10.84)	6.73 (6.58 to 6.88)	4.44 (4.28 to 4.61)		4.63 (4.47 to 4.80)	–*0.21 (–0.21 to –0.20)*		–*0.10 (–0.11 to –0.90)*		*0.11 (0.10 to 0.12)*	
	Unmarried	10.97 (10.87 to 11.07)	14.39 (14.12 to 14.66)	13.33 (13.11 to 13.54)	12.25 (12.04 to 12.45)	7.58 (7.37 to 7.79)	4.78 (4.57 to 4.99)		5.33 (5.11 to 5.55)	–*0.24 (–0.25 to –0.23)*		–*0.12 (–0.13 to –0.11)*		*0.12 (0.11 to 0.13)*	
**Region of residence**															
	Urban	10.11 (10.03 to 10.19)	13.73 (13.52 to 13.95)	12.41 (12.25 to 12.57)	10.86 (10.71 to 11.01)	6.65 (6.49 to 6.80)	4.25 (4.09 to 4.42)		4.36 (4.20 to 4.51)	–*0.22 (–0.23 to –0.21)*		–*0.12 (–0.13 to –0.12)*		*0.10 (0.09 to 0.11)*	
	Rural	10.85 (10.74 to 10.95)	13.71 (13.44 to 13.98)	12.32 (12.12 to 12.52)	12.03 (11.82 to 12.24)	7.90 (7.67 to 8.14)	5.30 (5.04 to 5.56)		6.15 (5.86 to 6.43)	–*0.21 (–0.22 to –0.21)*		–*0.08 (–0.09 to –0.07)*		*0.13 (0.12 to 0.14)*	
**Household income**															
	Unknown	7.62 (7.44 to 7.80)	12.26 (11.64 to 12.88)	11.54 (10.94 to 12.14)	9.32 (8.26 to 10.39)	6.96 (6.66 to 7.26)	4.42 (4.14 to 4.71)		5.05 (4.74 to 5.36)	–*0.20 (–0.21 to –0.18)*		–*0.09 (–0.11 to –0.08)*		*0.11 (0.09 to 0.13)*	
	Low (<3 million KRW^c^ per month)	12.21 (12.12 to 12.31)	15.90 (15.65 to 16.14)	13.82 (13.64 to 14.01)	12.20 (12.02 to 12.37)	7.84 (7.63 to 8.05)	5.57 (5.33 to 5.81)		5.96 (5.71 to 6.22)	–*0.25 (–0.25 to –0.24)*		–*0.80 (–0.90 to –0.70)*		*0.17 (0.16 to 0.18)*	
	Middle (3-5 million KRW per month)	10.10 (10.00 to 10.21)	12.21 (11.93 to 12.50)	11.74 (11.52 to 11.95)	10.83 (10.63 to 11.03)	7.12 (6.88 to 7.35)	4.29 (4.02 to 4.56)		4.52 (4.25 to 4.79)	–*0.15 (–0.16 to –0.14)*		–*0.13 (–0.14 to –0.12)*		*0.02 (0.01 to 0.03)*	
	High (≥5 million KRW per month)	8.53 (8.42 to 8.64)	11.04 (10.68 to 11.40)	10.81 (10.57 to 11.06)	10.13 (9.89 to 10.36)	6.24 (6.02 to 6.45)	3.89 (3.65 to 4.13)		4.18 (3.96 to 4.41)	–*0.16 (–0.16 to –0.15)*		–*0.12 (–0.13 to –0.11)*		*0.04 (–0.12 to 0.20)*	
**Basic livelihood security recipient**															
	Yes	19.07 (18.68 to 19.46)	28.24 (27.17 to 29.32)	22.80 (21.94 to 23.66)	19.64 (18.85 to 20.42)	14.31 (13.41 to 15.22)	8.83 (7.99 to 9.67)		10.00 (9.20 to 10.80)	–*0.43 (–0.46 to –0.40)*		–*0.20 (–0.24 to –0.16)*		*0.23 (0.18 to 0.28)*	
	No	10.08 (10.02 to 10.15)	13.30 (13.13 to 13.47)	12.12 (11.99 to 12.24)	11.00 (10.87 to 11.12)	6.82 (6.69 to 6.95)	4.41 (4.28 to 4.55)		4.69 (4.55 to 4.83)	–*0.21 (–0.21 to –0.20)*		–*0.10 (–0.11 to –0.10)*		*0.11 (0.10 to 0.12)*	
**Occupation**															
	White-collar	10.52 (10.41 to 10.63)	13.27 (12.96 to 13.58)	12.94 (12.70 to 13.18)	12.17 (11.94 to 12.40)	7.20 (6.98 to 7.43)	4.00 (3.77 to 4.23)		4.68 (4.44 to 4.92)	–*0.17 (–0.18 to –0.17)*		–*0.15 (–0.16 to –0.13)*		0.02 (0.01 to 0.04)	
	Blue-collar	11.36 (11.26 to 11.45)	15.15 (14.90 to 15.40)	13.45 (13.25 to 13.64)	12.24 (12.06 to 12.42)	7.75 (7.55 to 7.95)	5.01 (4.80 to 5.23)		5.40 (5.18 to 5.61)	–*0.22 (–0.23 to –0.22)*		–*0.10 (–0.11 to –0.09)*		*0.12 (0.11 to 0.13)*	
	Unemployed	9.15 (9.06 to 9.23)	12.64 (12.41 to 12.88)	10.88 (10.70 to 11.05)	9.42 (9.25 to 9.59)	6.14 (5.97 to 6.32)	4.57 (4.36 to 4.77)		4.55 (4.35 to 4.75)	–*0.23 (–0.24 to –0.22)*		–*0.08 (–0.09 to –0.07)*		*0.15 (0.14 to 0.16)*	
**Education**															
	Elementary school or lower	12.49 (12.35 to 12.62)	17.68 (17.33 to 18.04)	14.46 (14.19 to 14.73)	11.97 (11.72 to 12.23)	7.82 (7.54 to 8.10)	5.59 (5.26 to 5.92)		5.90 (5.57 to 6.23)	–*0.30 (–0.31 to –0.29)*		–*0.07 (–0.09 to –0.06)*		*0.23 (0.21 to 0.25)*	
	Middle school	10.27 (10.11 to 10.43)	14.48 (14.04 to 14.92)	11.98 (11.65 to 12.32)	10.09 (9.78 to 10.40)	7.10 (6.76 to 7.44)	4.73 (4.32 to 5.13)		5.00 (4.61 to 5.38)	–*0.22 (–0.23 to –0.21)*		–*0.08 (–0.10 to –0.07)*		*0.14 (0.12 to 0.16)*	
	High school	10.30 (10.20 to 10.40)	13.67 (13.40 to 13.94)	12.14 (11.94 to 12.35)	10.95 (10.76 to 11.15)	7.00 (6.79 to 7.21)	5.04 (4.79 to 5.29)		5.13 (4.89 to 5.37)	–*0.19 (–0.20 to –0.19)*		–*0.09 (–0.10 to –0.08)*		*0.10 (0.09 to 0.11)*	
	College or higher	9.79 (9.71 to 9.88)	12.19 (11.95 to 12.42)	12.00 (11.83 to 12.18)	11.38 (11.21 to 11.55)	6.85 (6.67 to 7.02)	4.07 (3.89 to 4.25)		4.54 (4.35 to 4.73)	–*0.16 (–0.16 to –0.15)*		–*0.13 (–0.14 to –0.12)*		*0.30 (0.20 to 0.40)*	
**Smoking status**															
	Nonsmoking	10.01 (9.94 to 10.07)	13.59 (13.40 to 13.77)	12.20 (12.06 to 12.33)	10.89 (10.76 to 11.02)	6.75 (6.62 to 6.89)	4.36 (4.22 to 4.50)		4.61 (4.46 to 4.75)	–*0.23 (–0.23 to –0.22)*		–*0.10 (–0.11 to –0.10)*		*0.13 (0.12 to 0.14)*	
	Smoking	11.52 (11.40 to 11.64)	14.13 (13.83 to 14.44)	13.00 (12.76 to 13.25)	12.48 (12.23 to 12.74)	8.11 (7.83 to 8.39)	5.53 (5.20 to 5.86)		6.18 (5.84 to 6.51)	–*0.17 (–0.18 to –0.16)*		–*0.09 (–0.11 to –0.08)*		*0.08 (0.06 to 0.10)*	
**Alcohol drink frequency**															
	No	10.41 (10.33 to 10.49)	13.92 (13.70 to 14.15)	12.51 (12.33 to 12.68)	10.96 (10.79 to 11.12)	6.81 (6.64 to 6.98)	4.91 (4.71 to 5.11)		5.11 (4.90 to 5.31)	–*0.24 (–0.24 to –0.23)*		–*0.09 (–0.10 to –0.08)*		*0.15 (0.14 to 0.16)*	
	Monthly	10.38 (10.29 to 10.48)	13.26 (13.00 to 13.51)	12.24 (12.05 to 12.44)	11.14 (10.95 to 11.33)	6.92 (6.72 to 7.12)	4.21 (3.97 to 4.45)		4.75 (4.51 to 4.99)	–*0.20 (–0.21 to –0.19)*		–*0.10 (–0.11 to –0.09)*		*0.10 (0.09 to 0.11)*	
	Weekly	10.13 (10.03 to 10.24)	14.10 (13.78 to 14.42)	12.38 (12.14 to 12.62)	11.79 (11.56 to 12.02)	7.57 (7.32 to 7.82)	4.42 (4.19 to 4.64)		4.79 (4.58 to 5.00)	–*0.19 (–0.20 to –0.18)*		–*0.13 (–0.14 to –0.12)*		*0.06 (0.05 to 0.07)*	
**Walking per week**															
	Rarely	11.79 (11.66 to 11.93)	14.46 (14.12 to 14.80)	13.55 (13.29 to 13.81)	12.50 (12.24 to 12.76)	8.28 (7.98 to 8.58)	6.03 (5.70 to 6.35)		6.25 (5.90 to 6.61)	–*0.21 (–0.22 to –0.20)*		–*0.09 (–0.10 to –0.07)*		*0.12 (0.10 to 0.14)*	
	1-2 times	11.52 (11.36 to 11.67)	15.76 (15.29 to 16.22)	13.70 (13.38 to 14.02)	12.51 (12.20 to 12.82)	8.26 (7.94 to 8.59)	4.94 (4.59 to 5.29)		5.67 (5.30 to 6.04)	–*0.25 (–0.26 to –0.23)*		–*0.13 (–0.15 to –0.11)*		*0.12 (0.10 to 0.15)*	
	3-4 times	9.72 (9.59 to 9.84)	13.47 (13.09 to 13.84)	11.74 (11.48 to 12.01)	10.56 (10.31 to 10.81)	6.52 (6.27 to 6.78)	4.48 (4.20 to 4.76)		4.82 (4.54 to 5.11)	–*0.23 (–0.24 to –0.22)*		–*0.08 (–0.10 to –0.07)*		*0.15 (0.13 to 0.17)*	
	≥5 times	9.81 (9.73 to 9.89)	13.19 (12.98 to 13.41)	11.87 (11.70 to 12.03)	10.71 (10.56 to 10.87)	6.57 (6.41 to 6.73)	4.09 (3.92 to 4.26)		4.45 (4.28 to 4.62)	–*0.21 (–0.22 to –0.21)*		–*0.10 (–0.11 to –0.10)*		*0.11 (0.10 to 0.12)*	
**Subjective health status**															
	Good	6.92 (6.85 to 7.00)	9.06 (8.86 to 9.25)	8.34 (8.19 to 8.49)	7.76 (7.61 to 7.91)	4.45 (4.30 to 4.61)	2.92 (2.76 to 3.07)		3.13 (2.97 to 3.29)	–*0.13 (–0.14 to –0.13)*		–*–0.06 (–0.07 to –0.06)*		*0.07 (0.06 to 0.08)*	
	Normal	11.44 (11.35 to 11.52)	15.58 (15.32 to 15.83)	13.66 (13.47 to 13.85)	12.53 (12.36 to 12.71)	7.65 (7.47 to 7.83)	5.02 (4.81 to 5.23)		5.48 (5.26 to 5.70)	–*0.24 (–0.24 to –0.23)*		–*0.11 (–0.12 to –0.10)*		*0.13 (0.12 to 0.14)*	
	Bad	17.30 (17.14 to 17.46)	23.27 (22.84 to 23.71)	20.32 (20.00 to 20.65)	17.28 (16.97 to 17.60)	12.09 (11.75 to 12.44)	9.27 (8.82 to 9.73)		9.32 (8.89 to 9.75)	–*0.36 (–0.37 to –0.35)*		–*0.11 (–0.13 to –0.09)*		*0.25 (0.23 to 0.27)*	
**Depression**															
	No	9.36 (9.30 to 9.42)	12.41 (12.25 to 12.58)	11.31 (11.18 to 11.43)	10.20 (10.08 to 10.31)	6.32 (6.19 to 6.45)	4.02 (3.88 to 4.15)		4.21 (4.08 to 4.34)	–*0.20 (–0.20 to –0.19)*		–*0.10 (–0.11 to –0.10)*		*0.10 (0.09 to 0.11)*	
	Yes	24.43 (24.14 to 24.72)	32.54 (31.78 to 33.29)	28.73 (28.12 to 29.34)	26.24 (25.66 to 26.82)	17.97 (17.31 to 18.62)	11.69 (11.00 to 12.39)		13.46 (12.76 to 14.17)	–*0.46 (–0.49 to –0.44)*		–*0.23 (–0.27 to –0.20)*		*0.23 (0.19 to 0.27)*	
**Hypertension**															
	No	9.25 (9.14 to 9.35)	14.27 (13.93 to 14.61)	11.46 (11.22 to 11.69)	9.56 (9.35 to 9.77)	6.28 (6.06 to 6.49)	4.06 (3.84 to 4.29)		4.56 (4.33 to 4.79)	–*0.27 (–0.28 to –0.26)*		–*0.08 (–0.09 to –0.07)*		*0.19 (0.18 to 0.20)*	
	Yes	10.59 (10.52 to 10.66)	13.62 (13.44 to 13.80)	12.59 (12.45 to 12.73)	11.62 (11.49 to 11.76)	7.21 (7.06 to 7.36)	4.70 (4.55 to 4.86)		4.99 (4.83 to 5.15)	–*0.20 (–0.20 to –0.19)*		–*0.11 (–0.12 to –0.11)*		*0.09 (0.08 to 0.10)*	
**Diabetes mellitus**															
	No	9.26 (9.10 to 9.42)	14.85 (14.30 to 15.39)	11.78 (11.41 to 12.15)	9.70 (9.38 to 10.02)	6.41 (6.08 to 6.74)	3.95 (3.63 to 4.27)		4.25 (3.93 to 4.57)	–*0.28 (–0.29 to –0.27)*		–*0.10 (–0.11 to –0.08)*		*0.18 (0.16 to 0.20)*	
	Yes	10.42 (10.36 to 10.49)	13.65 (13.48 to 13.83)	12.43 (12.30 to 12.56)	11.35 (11.22 to 11.47)	7.08 (6.94 to 7.21)	4.63 (4.48 to 4.77)		4.96 (4.81 to 5.11)	–*0.21 (–0.21 to –0.20)*		–*0.10 (–0.11 to –0.10)*		*0.11 (0.10 to 0.12)*	
**BMI groups**															
	Underweight (<18.5 kg/m^2^)	12.94 (12.73 to 13.15)	16.11 (15.57 to 16.64)	15.50 (15.08 to 15.91)	13.67 (13.24 to 14.11)	7.55 (7.11 to 7.99)	5.53 (4.90 to 6.16)		5.61 (5.02 to 6.20)	–*0.29 (–0.30 to –0.27)*		–*0.10 (–0.13 to –0.07)*		*0.19 (0.16 to 0.22)*	
	Normal (18.5-22.9 kg/m^2^)	10.59 (10.50 to 10.68)	13.69 (13.46 to 13.91)	12.44 (12.27 to 12.62)	11.42 (11.25 to 11.59)	7.18 (6.99 to 7.37)	4.53 (4.33 to 4.72)		4.89 (4.69 to 5.10)	–*0.20 (–0.21 to –0.19)*		–*0.11 (–0.12 to –0.10)*		*0.09 (0.08 to 0.10)*	
	Overweight (23.0-24.9 kg/m^2^)	9.67 (9.57 to 9.78)	13.08 (12.78 to 13.38)	11.51 (11.29 to 11.73)	10.40 (10.19 to 10.62)	6.62 (6.40 to 6.84)	4.47 (4.22 to 4.72)		4.76 (4.51 to 5.01)	–*0.20 (–0.21 to –0.19)*		–*0.09 (–0.10 to –0.08)*		*0.11 (0.10 to 0.12)*	
	Obese (≥25.0 kg/m^2^)	9.89 (9.79 to 9.99)	13.70 (13.41 to 14.00)	12.18 (11.96 to 12.40)	11.04 (10.84 to 11.25)	7.03 (6.84 to 7.23)	4.55 (4.33 to 4.77)		4.88 (4.66 to 5.10)	–*0.22 (–0.22 to –0.21)*		–*0.10 (–0.11 to –0.09)*		*0.12 (0.11 to 0.13)*	

^a^Values in italics indicate statistical significance (*P*<.05).

^b^The β-coefficient was multiplied by 10. The estimated β (95% CI) was derived using a weighted linear regression model.

^c^1 KRW=US $0.00073.

**Table 3:** Prevalence and trends for unmet dental care needs.^a^

Characteristics		Total, weighted percentage (95% CI)	Before the pandemic, weighted percentage (95% CI)					During the pandemic, weighted percentage (95% CI)			Trends before the pandemic, β-coefficient (95% CI)^b^		Trends during the pandemic, β-coefficient (95% CI)^b^		Difference in trends, β-coefficient (95% CI)^b^
			2009-2011	2012-2014	2015-2017	2018-2019	2021		2022						
Overall		20.35 (20.27 to 20.43)	25.13 (24.90 to 25.35)	23.53 (23.35 to 23.70)	20.81 (20.64 to 20.97)	15.51 (15.32 to 15.69)	13.70 (13.47 to 13.93)		13.56 (13.33 to 13.78)	–*0.32 (–0.32 to –0.31)*		–*0.09 (–0.10 to –0.09)*		*0.23 (0.22 to 0.24)*	
**Age (years)**															
	19-39	20.75 (20.62 to 20.89)	24.74 (24.41 to 25.07)	24.03 (23.77 to 24.30)	21.90 (21.63 to 22.16)	15.21 (14.91 to 15.50)	11.91 (11.54 to 12.27)		12.12 (11.74 to 12.51)	–*0.26 (–0.27 to –0.25)*		–*0.19 (–0.20 to –0.17)*		*0.07 (0.05 to 0.09)*	
	40-59	21.27 (21.15 to 21.39)	25.46 (25.14 to 25.77)	24.09 (23.85 to 24.33)	21.61 (21.38 to 21.84)	16.66 (16.38 to 16.93)	15.66 (15.29 to 16.02)		15.27 (14.91 to 15.64)	–*0.24 (–0.25 to –0.23)*		–*0.06 (–0.08 to –0.05)*		*0.18 (0.16 to 0.20)*	
	≥60	18.15 (18.03 to 18.28)	25.27 (24.89 to 25.66)	21.53 (21.25 to 21.80)	17.80 (17.55 to 18.04)	14.10 (13.84 to 14.36)	13.07 (12.73 to 13.41)		12.84 (12.53 to 13.16)	–*0.32 (–0.32 to –0.31)*		–*0.05 (–0.06 to –0.04)*		*0.27 (0.26 to 0.28)*	
**Sex**															
	Men	19.04 (18.93 to 19.14)	23.19 (22.91 to 23.46)	22.01 (21.79 to 22.23)	19.58 (19.37 to 19.79)	14.63 (14.39 to 14.86)	12.68 (12.39 to 12.97)		12.84 (12.55 to 13.13)	–*0.25 (–0.26 to –0.25)*		–*0.08 (–0.09 to –0.07)*		*0.17 (0.16 to 0.18)*	
	Women	21.65 (21.54 to 21.76)	27.04 (26.76 to 27.32)	25.02 (24.81 to 25.24)	22.02 (21.82 to 22.22)	16.39 (16.15 to 16.62)	14.70 (14.40 to 15.00)		14.26 (13.97 to 14.55)	–*0.30 (–0.31 to –0.30)*		–*0.09 (–0.11 to –0.08)*		*0.21 (0.19 to 0.23)*	
**Marriage**															
	Married	20.40 (20.30 to 20.49)	25.18 (24.92 to 25.43)	23.52 (23.32 to 23.71)	20.69 (20.51 to 20.88)	15.48 (15.26 to 15.69)	13.84 (13.56 to 14.12)		13.36 (13.08 to 13.63)	–*0.28 (–0.29 to –0.28)*		–*0.09 (–0.10 to –0.08)*		*0.19 (0.18 to 0.20)*	
	Unmarried	20.26 (20.13 to 20.39)	25.02 (24.67 to 25.38)	23.54 (23.27 to 23.81)	21.04 (20.78 to 21.29)	15.57 (15.28 to 15.85)	13.44 (13.10 to 13.78)		13.90 (13.55 to 14.25)	–*0.29 (–0.30 to –0.28)*		–*0.09 (–0.11 to –0.07)*		*0.20 (0.18 to 0.22)*	
**Region of residence**															
	Urban	20.21 (20.11 to 20.32)	25.68 (25.39 to 25.96)	23.83 (23.62 to 24.04)	20.41 (20.21 to 20.61)	14.94 (14.71 to 15.16)	13.17 (12.88 to 13.45)		12.86 (12.59 to 13.14)	–*0.35 (–0.36 to –0.34)*		–*0.08 (–0.09 to –0.07)*		*0.27 (0.26 to 0.28)*	
	Rural	20.66 (20.53 to 20.80)	23.90 (23.55 to 24.25)	22.83 (22.55 to 23.10)	21.72 (21.45 to 21.98)	16.85 (16.54 to 17.16)	14.94 (14.56 to 15.33)		15.19 (14.79 to 15.59)	–*0.22 (–0.23 to –0.21)*		–*0.09 (–0.11 to –0.08)*		*0.13 (0.11 to 0.15)*	
**Household income**															
	Unknown	16.54 (16.28 to 16.79)	21.82 (21.02 to 22.62)	22.40 (21.57 to 23.23)	17.52 (16.11 to 18.93)	14.98 (14.57 to 15.39)	13.38 (12.91 to 13.86)		13.68 (13.17 to 14.20)	–*0.21 (–0.23 to –0.19)*		–*0.06 (–0.08 to –0.04)*		*0.15 (0.12 to 0.18)*	
	Low (<3 million KRW^c^ per month)	23.62 (23.49 to 23.74)	28.50 (28.20 to 28.81)	26.37 (26.13 to 26.61)	22.79 (22.57 to 23.02)	17.56 (17.26 to 17.86)	15.86 (15.47 to 16.26)		16.07 (15.68 to 16.47)	–*0.30 (–0.31 to –0.30)*		–*0.06 (–0.08 to –0.05)*		*0.24 (0.22 to 0.26)*	
	Middle (3-5 million KRW per month)	20.19 (20.04 to 20.33)	23.53 (23.15 to 23.91)	22.76 (22.48 to 23.05)	20.30 (20.03 to 20.56)	15.92 (15.57 to 16.26)	13.73 (13.26 to 14.20)		13.47 (13.01 to 13.93)	–*0.23 (–0.24 to –0.22)*		–*0.10 (–0.12 to –0.08)*		*0.13 (0.11 to 0.15)*	
	High (≥5 million KRW per month)	16.69 (16.54 to 16.84)	20.12 (19.65 to 20.59)	19.67 (19.35 to 19.99)	18.18 (17.88 to 18.49)	13.60 (13.29 to 13.91)	11.80 (11.40 to 12.20)		11.52 (11.16 to 11.88)	–*0.21 (–0.22 to –0.20)*		–*0.11 (–0.13 to –0.10)*		*0.10 (0.08 to 0.12)*	
**Basic livelihood security recipient**															
	Yes	33.99 (33.52 to 34.46)	43.94 (42.75 to 45.14)	38.61 (37.61 to 39.62)	33.46 (32.52 to 34.41)	27.97 (26.83 to 29.12)	23.67 (22.38 to 24.97)		26.15 (24.91 to 27.38)	–*0.41 (–0.44 to –0.38)*		–*0.13 (–0.18 to –0.07)*		*0.28 (0.22 to 0.34)*	
	No	19.96 (19.88 to 20.05)	24.58 (24.35 to 24.80)	23.14 (22.97 to 23.31)	20.47 (20.31 to 20.64)	15.17 (14.98 to 15.35)	13.34 (13.11 to 13.58)		13.07 (12.84 to 13.30)	–*0.27 (–0.28 to –0.27)*		–*0.09 (–0.10 to –0.08)*		*0.18 (0.17 to 0.19)*	
**Occupation**															
	White-collar	19.14 (18.99 to 19.28)	23.08 (22.68 to 23.47)	22.46 (22.15 to 22.76)	20.55 (20.26 to 20.84)	14.67 (14.34 to 14.99)	12.31 (11.92 to 12.70)		11.61 (11.23 to 11.98)	–*0.25 (–0.26 to –0.24)*		–*0.15 (–0.17 to –0.13)*		*0.10 (0.08 to 0.12)*	
	Blue-collar	22.36 (22.24 to 22.49)	26.96 (26.64 to 27.28)	25.61 (25.36 to 25.86)	22.84 (22.60 to 23.07)	17.40 (17.12 to 17.68)	15.39 (15.03 to 15.75)		15.44 (15.09 to 15.79)	–*0.26 (–0.27 to –0.25)*		–*0.08 (–0.09 to –0.06)*		*0.18 (0.16 to 0.20)*	
	Unemployed	19.17 (19.05 to 19.29)	24.68 (24.37 to 24.99)	22.12 (21.88 to 22.36)	18.85 (18.62 to 19.08)	14.20 (13.95 to 14.46)	13.12 (12.78 to 13.46)		13.23 (12.89 to 13.57)	–*0.32 (–0.33 to –0.31)*		–*0.06 (–0.08 to –0.05)*		*0.26 (0.24 to 0.28)*	
**Education**															
	Elementary school or lower	22.93 (22.76 to 23.11)	29.25 (28.82 to 29.69)	25.59 (25.25 to 25.93)	21.60 (21.27 to 21.93)	17.46 (17.07 to 17.85)	15.48 (14.93 to 16.03)		15.66 (15.14 to 16.18)	–*0.31 (–0.32 to –0.30)*		–*0.07 (–0.09 to –0.05)*		*0.24 (0.22 to 0.26)*	
	Middle school	21.49 (21.27 to 21.71)	27.10 (26.53 to 27.66)	24.19 (23.75 to 24.64)	20.74 (20.32 to 21.17)	16.61 (16.11 to 17.11)	14.89 (14.23 to 15.56)		15.45 (14.80 to 16.10)	–*0.29 (–0.30 to –0.27)*		–*0.05 (–0.08 to –0.03)*		*0.24 (0.21 to 0.27)*	
	High school	21.74 (21.60 to 21.88)	26.24 (25.89 to 26.59)	24.94 (24.66 to 25.22)	21.95 (21.68 to 22.22)	16.72 (16.42 to 17.03)	15.44 (15.03 to 15.85)		15.22 (14.83 to 15.61)	–*0.27 (–0.28 to –0.27)*		–*0.07 (–0.08 to –0.05)*		*0.20 (0.18 to 0.22)*	
	College or higher	18.60 (18.48 to 18.71)	22.40 (22.09 to 22.71)	21.88 (21.64 to 22.11)	19.93 (19.71 to 20.15)	14.18 (13.94 to 14.43)	12.20 (11.90 to 12.50)		11.93 (11.63 to 12.23)	–*0.25 (–0.26 to –0.24)*		–*0.11 (–0.13 to –0.10)*		*0.14 (0.02 to 0.26)*	
**Smoking status**															
	Nonsmoking	19.42 (19.33 to 19.51)	24.49 (24.24 to 24.73)	22.67 (22.49 to 22.86)	19.91 (19.74 to 20.08)	14.69 (14.49 to 14.88)	13.00 (12.76 to 13.24)		12.67 (12.43 to 12.91)	–*0.29 (–0.29 to –0.28)*		–*0.09 (–0.10 to –0.08)*		*0.20 (0.19 to 0.21)*	
	Smoking	23.76 (23.59 to 23.92)	27.06 (26.67 to 27.45)	26.35 (26.02 to 26.68)	24.23 (23.90 to 24.56)	18.86 (18.47 to 19.26)	16.97 (16.44 to 17.50)		17.57 (17.04 to 18.10)	–*0.22 (–0.23 to –0.21)*		–*0.05 (–0.07 to –0.03)*		*0.17 (0.15 to 0.19)*	
**Alcohol drink frequency**															
	No	20.40 (20.29 to 20.52)	25.14 (24.85 to 25.44)	23.42 (23.19 to 23.65)	20.48 (20.26 to 20.69)	15.28 (15.04 to 15.53)	14.49 (14.16 to 14.82)		14.26 (13.93 to 14.60)	–*0.28 (–0.29 to –0.28)*		–*0.05 (–0.07 to –0.04)*		*0.23 (0.21 to 0.25)*	
	Monthly	19.84 (19.71 to 19.97)	24.14 (23.81 to 24.47)	22.83 (22.57 to 23.08)	20.01 (19.77 to 20.25)	14.59 (14.32 to 14.87)	13.15 (12.74 to 13.56)		12.91 (12.53 to 13.29)	–*0.29 (–0.30 to –0.28)*		–*0.06 (–0.08 to –0.05)*		*0.23 (0.21 to 0.25)*	
	Weekly	20.96 (20.82 to 21.11)	26.71 (26.30 to 27.12)	24.81 (24.49 to 25.12)	22.59 (22.29 to 22.90)	17.29 (16.94 to 17.63)	13.17 (12.81 to 13.52)		13.36 (13.00 to 13.71)	–*0.26 (–0.28 to –0.25)*		–*0.18 (–0.20 to –0.17)*		*0.08 (0.06 to 0.10)*	
**Walking per week**															
	Rarely	21.95 (21.77 to 22.12)	25.18 (24.74 to 25.62)	24.34 (24.00 to 24.67)	22.20 (21.87 to 22.53)	17.33 (16.93 to 17.73)	15.89 (15.37 to 16.41)		16.70 (16.14 to 17.26)	–*0.24 (–0.25 to –0.23)*		–*0.03 (–0.05 to –0.01)*		*0.21 (0.19 to 0.23)*	
	1-2 times	22.86 (22.66 to 23.07)	28.64 (28.07 to 29.22)	26.21 (25.80 to 26.62)	23.53 (23.13 to 23.93)	18.17 (17.72 to 18.63)	15.29 (14.73 to 15.85)		15.29 (14.69 to 15.90)	–*0.32 (–0.34 to –0.31)*		–*0.15 (–0.17 to –0.12)*		*0.17 (0.14 to 0.20)*	
	3-4 times	20.33 (20.16 to 20.51)	25.73 (25.25 to 26.21)	23.66 (23.30 to 24.02)	20.84 (20.51 to 21.18)	15.66 (15.27 to 16.04)	13.78 (13.32 to 14.25)		13.59 (13.11 to 14.07)	–*0.31 (–0.33 to –0.30)*		–*0.08 (–0.10 to –0.06)*		*0.23 (0.21 to 0.26)*	
	≥5 times	19.31 (19.20 to 19.42)	24.30 (24.02 to 24.58)	22.56 (22.34 to 22.79)	19.72 (19.51 to 19.92)	14.41 (14.18 to 14.64)	12.64 (12.34 to 12.93)		12.49 (12.22 to 12.77)	–*0.29 (–0.29 to –0.28)*		–*0.09 (–0.10 to –0.08)*		*0.20 (0.19 to 0.21)*	
**Subjective health status**															
	Good	15.76 (15.66 to 15.87)	18.96 (18.69 to 19.23)	18.56 (18.34 to 18.78)	16.51 (16.30 to 16.72)	11.27 (11.04 to 11.51)	10.56 (10.28 to 10.84)		10.20 (9.92 to 10.48)	–*0.21 (–0.22 to –0.21)*		–*0.05 (–0.06 to –0.03)*		*0.16 (0.14 to 0.18)*	
	Normal	22.40 (22.28 to 22.52)	28.45 (28.12 to 28.77)	25.82 (25.58 to 26.06)	22.87 (22.64 to 23.10)	16.98 (16.72 to 17.24)	15.14 (14.80 to 15.48)		15.15 (14.82 to 15.49)	–*0.33 (–0.34 to –0.32)*		–*0.08 (–0.10 to –0.07)*		*0.25 (0.23 to 0.27)*	
	Bad	28.04 (27.85 to 28.23)	35.36 (34.87 to 35.86)	31.21 (30.83 to 31.59)	27.06 (26.70 to 27.43)	22.46 (22.03 to 22.89)	20.60 (19.97 to 21.23)		20.50 (19.91 to 21.09)	–*0.36 (–0.37 to –0.34)*		–*0.08 (–0.10 to –0.06)*		*0.28 (0.26 to 0.31)*	
**Depression**															
	No	19.30 (19.21 to 19.38)	23.70 (23.47 to 23.92)	22.43 (22.26 to 22.61)	19.80 (19.64 to 19.96)	14.62 (14.44 to 14.81)	12.88 (12.65 to 13.11)		12.61 (12.39 to 12.84)	–*0.27 (–0.27 to –0.26)*		–*0.09 (–0.10 to –0.08)*		*0.18 (0.17 to 0.19)*	
	Yes	35.67 (35.35 to 35.99)	45.60 (44.80 to 46.40)	40.09 (39.43 to 40.76)	35.61 (34.98 to 36.24)	29.31 (28.55 to 30.08)	24.28 (23.36 to 25.21)		25.41 (24.52 to 26.30)	–*0.50 (–0.53 to –0.48)*		–*0.21 (–0.25 to –0.17)*		*0.29 (0.24 to 0.34)*	
**Hypertension**															
	No	20.03 (19.88 to 20.18)	26.46 (26.03 to 26.90)	23.43 (23.11 to 23.75)	19.87 (19.58 to 20.16)	15.54 (15.22 to 15.86)	14.32 (13.91 to 14.74)		14.59 (14.18 to 14.99)	–*0.32 (–0.33 to –0.31)*		–*0.05 (–0.07 to –0.03)*		*0.27 (0.25 to 0.29)*	
	Yes	20.43 (20.33 to 20.52)	24.87 (24.63 to 25.11)	23.55 (23.36 to 23.73)	21.04 (20.86 to 21.21)	15.50 (15.29 to 15.70)	13.53 (13.27 to 13.78)		13.26 (13.00 to 13.51)	–*0.27 (–0.27 to –0.26)*		–*0.10 (–0.11 to –0.09)*		*0.17 (0.16 to 0.18)*	
**Diabetes mellitus**															
	No	20.90 (20.67 to 21.12)	28.38 (27.69 to 29.08)	24.50 (24.00 to 25.01)	20.91 (20.47 to 21.35)	16.63 (16.14 to 17.13)	15.05 (14.43 to 15.66)		14.86 (14.28 to 15.44)	–*0.34 (–0.36 to –0.32)*		–*0.07 (–0.10 to –0.05)*		*0.27 (0.24 to 0.30)*	
	Yes	20.30 (20.22 to 20.39)	24.92 (24.69 to 25.15)	23.45 (23.28 to 23.63)	20.80 (20.63 to 20.96)	15.41 (15.22 to 15.60)	13.56 (13.32 to 13.80)		13.41 (13.17 to 13.65)	–*0.28 (–0.28 to –0.27)*		–*0.09 (–0.10 to –0.08)*		*0.19 (0.18 to 0.20)*	
**BMI groups**															
	Underweight (<18.5 kg/m^2^)	22.10 (21.84 to 22.37)	26.50 (25.83 to 27.18)	25.08 (24.58 to 25.58)	22.13 (21.61 to 22.65)	16.09 (15.46 to 16.72)	14.54 (13.61 to 15.47)		13.58 (12.67 to 14.49)	–*0.31 (–0.33 to –0.29)*		–*0.10 (–0.14 to –0.06)*		*0.21 (0.17 to 0.25)*	
	Normal (18.5-22.9 kg/m^2^)	20.31 (20.19 to 20.42)	24.90 (24.60 to 25.19)	23.17 (22.95 to 23.40)	20.67 (20.45 to 20.89)	15.15 (14.89 to 15.41)	13.22 (12.90 to 13.54)		13.19 (12.87 to 13.51)	–*0.27 (–0.28 to –0.26)*		–*0.09 (–0.10 to –0.07)*		*0.18 (0.16 to 0.20)*	
	Overweight (23.0-24.9 kg/m^2^)	19.55 (19.41 to 19.70)	24.16 (23.78 to 24.54)	22.81 (22.51 to 23.11)	19.80 (19.52 to 20.08)	14.90 (14.59 to 15.22)	13.31 (12.89 to 13.72)		13.26 (12.87 to 13.66)	–*0.27 (–0.28 to –0.26)*		–*0.07 (–0.09 to –0.06)*		*0.20 (0.18 to 0.22)*	
	Obese (≥25.0 kg/m^2^)	20.68 (20.55 to 20.82)	26.12 (25.73 to 26.50)	24.36 (24.06 to 24.65)	21.58 (21.31 to 21.85)	16.26 (15.97 to 16.55)	14.49 (14.11 to 14.86)		14.23 (13.86 to 14.60)	–*0.30 (–0.31 to –0.29)*		–*0.09 (–0.10 to –0.07)*		*0.21 (0.19 to 0.23)*	

^a^Values in italics indicate statistical significance (*P*<.05).

^b^The β-coefficient was multiplied by 10. Estimated β (95% CI) was derived using a weighted linear regression model.

^c^1 KRW=US $0.00073.

**Table 4:** Risk factors for unmet health care needs before and during the COVID-19 pandemic.^a^

Risk factor		Unmet health care needs before the pandemic, prevalence ratio (95% CI)	Unmet health care needs during the pandemic, prevalence ratio (95% CI)
**Age (years)**			
	≥60	1.00 (reference)	1.00 (reference)
	19-39	*1.07 (1.06-1.08)*	*1.01 (1.00-1.02)*
	40-59	*1.03 (1.03* *-* *1.04)*	*1.00 (1.00* *-* *1.01)*
**Sex**			
	Men	1.00 (reference)	1.00 (reference)
	Women	*1.04 (1.03* *-* *1.04)*	*1.02 (1.01* *-* *1.02)*
**Marriage**			
	Married	1.00 (reference)	1.00 (reference)
	Unmarried	*1.01 (1.01* *-* *1.02)*	*1.01 (1.00* *-* *1.01)*
**Region of residence**			
	Urban	1.00 (reference)	1.00 (reference)
	Rural	*1.01 (1.00-1.01)*	*1.01 (1.01* *-* *1.02)*
**Household income**			
	High (≥5 million KRW^b^ per month)	1.00 (reference)	1.00 (reference)
	Low (<3 million KRW per month)	0.99 (0.98-1.00)	*1.01 (1.01-1.02)*
	Middle (3-5 million KRW per month)	*1.00 (0.99-1.01)*	*1.01 (1.00-1.02)*
**Basic livelihood security recipients**			
	No	1.00 (reference)	1.00 (reference)
	Yes	*1.13 (1.12-1.15)*	*1.05 (1.04-1.07)*
**Occupation**			
	White-collar	1.00 (reference)	1.00 (reference)
	Blue-collar	*0.98 (0.98-0.98)*	*1.00 (1.00-1.01)*
	Unemployed	*0.96 (0.96-0.97)*	*1.00 (1.00-1.01)*
**Education**			
	College degree or higher	1.00 (reference)	1.00 (reference)
	Elementary school or lower	*1.09 (1.08-1.10)*	*1.05 (1.03-1.06)*
	Middle school	*1.06 (1.05-1.07)*	*1.03 (1.02-1.04)*
	High school	*1.03 (1.02-1.04)*	*1.02 (1.00-1.03)*
**Smoking status**			
	Nonsmoking	1.00 (reference)	1.00 (reference)
	Smoking	*1.01 (1.01-1.02)*	*1.01 (1.01-1.02)*
**Alcohol consumption frequency**			
	Weekly	1.00 (reference)	1.00 (reference)
	Rarely	0.99 (0.99-1.00)	*1.01 (1.00-1.01)*
	Monthly	1.00 (0.99-1.00)	*1.00 (1.00-1.01)*
**Walking per week**			
	≥5 times	1.00 (reference)	1.00 (reference)
	Rarely	*1.06 (1.06-1.07)*	*1.06 (1.05-1.07)*
	1-2 times	*1.04 (1.03-1.05)*	*1.04 (1.03-1.05)*
	3-4 times	*1.02 (1.01-1.03)*	*1.02 (1.01-1.03)*
**Subjective health status**			
	Good	1.00 (reference)	1.00 (reference)
	Normal	*1.13 (1.13-1.14)*	*1.07 (1.06-1.08)*
	Bad	*1.28 (1.27-1.29)*	*1.14 (1.13-1.16)*
**Depression**			
	No	1.00 (reference)	1.00 (reference)
	Yes	*1.23 (1.22-1.23)*	*1.10 (1.09-1.10)*
**Hypertension**			
	No	1.00 (reference)	1.00 (reference)
	Yes	*1.01 (1.01-1.02)*	*1.01 (1.00-1.01)*
**Diabetes mellitus**			
	No	1.00 (reference)	1.00 (reference)
	Yes	*0.99 (0.98-0.99)*	0.99 (0.99-1.00)
**BMI (kg/m^2^) groups**			
	Obese (≥25.0 kg/m^2^)	1.00 (reference)	1.00 (reference)
	Underweight (<18.5 kg/m^2^)	*1.10 (1.09-1.12)*	*1.03 (1.01-1.05)*
	Normal (18.5-22.9 kg/m^2^)	*1.07 (1.05-1.08)*	1.02 (0.99-1.04)
	Overweight (23.0-24.9 kg/m^2^)	*1.03 (1.02-1.05)*	1.01 (0.98-1.04)

^a^Values in italics indicate statistical significance (*P*<.05).

^b^1 KRW=US $0.00073.

**Table 5:** Risk factors for unmet dental care needs before and during the COVID-19 pandemic.^a^

Risk factor			Unmet dental care needs before the pandemic, prevalence ratio (95% CI)		Unmet dental care needs during the pandemic, prevalence ratio (95% CI)		
**Age (years)**							
	≥60	1.00 (reference)		1.00 (reference)			
	19-39	*1.07 (1.06-1.08)*		*0.98 (0.97-0.99)*			
	40-59	*1.03 (1.02-1.04)*		0.99 (0.98-1.00)			
**Sex**							
	Men	1.00 (reference)		1.00 (reference)			
	Women	*1.04 (1.03-1.04)*		*1.02 (1.01-1.03)*			
**Marriage**							
	Married	1.00 (reference)		1.00 (reference)			
	Unmarried	*1.00 (1.00-1.01)*		1.00 (0.99-1.01)			
**Region of residence**							
	Urban	1.00 (reference)		1.00 (reference)			
	Rural	*1.00 (1.00-1.01)*		*1.02 (1.01-1.03)*			
**Household income**							
	High (≥5 million KRW^b^ per month)	1.00 (reference)		1.00 (reference)			
	Low (<3 million KRW per month)	*1.01 (1.00-1.03)*		*0.98 (0.97-0.99)*			
	Middle (3-5 million KRW per month)	1.00 (0.99-1.02)		*0.96 (0.94-0.97)*			
**Basic livelihood security recipients**							
	No	1.00 (reference)		1.00 (reference)			
	Yes	*1.24 (1.21-1.26)*		*1.16 (1.13-1.19)*			
**Occupation**							
	White-collar	1.00 (reference)		1.00 (reference)			
	Blue-collar	1.00 (0.99-1.00)		*1.01 (1.01-1.02)*			
	Unemployed	0.99 (0.98-1.00)		*1.03 (1.02-1.04)*			
**Education**							
	College education or higher	1.00 (reference)		1.00 (reference)			
	Elementary or lower	*1.17 (1.15-1.19)*		*1.13 (1.11-1.15)*			
	Middle school	*1.11 (1.09-1.13)*		*1.08 (1.06-1.11)*			
	High school	*1.05 (1.04-1.07)*		*1.04 (1.02-1.07)*			
**Smoking status**							
	Nonsmoking	1.00 (reference)		1.00 (reference)			
	Smoking	*1.05 (1.05-1.06)*		*1.05 (1.05-1.06)*			
**Alcohol consumption frequency**							
	Weekly	1.00 (reference)		1.00 (reference)			
	Rarely	*0.96 (0.95-0.97)*		*1.03 (1.01-1.04)*			
	Monthly	*0.98 (0.97-0.99)*		*1.01 (1.00-1.03)*			
**Walking per week**							
	≥5 times	1.00 (reference)		1.00 (reference)			
	Rarely	*1.09 (1.08-1.10)*		*1.14 (1.12-1.16)*			
	1-2 times	*1.06 (1.05-1.07)*		*1.09 (1.07-1.12)*			
	3-4 times	*1.03 (1.01-1.04)*		*1.04 (1.02-1.07)*			
**Subjective health status**							
	Good	1.00 (reference)		1.00 (reference)			
	Normal	*1.17 (1.17-1.18)*		*1.13 (1.11-1.14)*			
	Bad	*1.38 (1.36-1.40)*		*1.27 (1.25-1.30)*			
**Depression**							
	No	1.00 (reference)		1.00 (reference)			
	Yes	*1.28 (1.27-1.29)*		*1.16 (1.15-1.17)*			
**Hypertension**							
	No	1.00 (reference)		1.00 (reference)			
	Yes	*1.01 (1.00-1.01)*		0.99 (0.98-1.00)			
**Diabetes mellitus**							
	No	1.00 (reference)		1.00 (reference)			
	Yes	*1.01 (1.00-1.02)*		*1.02 (1.01-1.03)*			
**BMI (kg/m^2^) groups**							
	Obese (≥25.0 kg/m^2^)	1.00 (reference)		1.00 (reference)			
	Underweight (<18.5 kg/m^2^)	*1.04 (1.02-1.06)*		0.99 (0.96-1.02)			
	Normal (18.5-22.9 kg/m^2^)	*1.03 (1.01-1.05)*		0.99 (0.96-1.03)			
	Overweight (23.0-24.9 kg/m^2^)	1.01 (0.99-1.04)		1.00 (0.96-1.04)			

^a^Values in italics indicate statistical significance (*P*<.05).

^b^1 KRW=US $0.00073.

## Discussion

### Principal Findings

Our study represents the first national analysis of unmet health care and dental care needs in South Korea using long-term, large-scale data from the KCHS. We assessed the prevalence of unmet health care and dental care needs from 2009 to 2022, focusing on trends before and during the COVID-19 pandemic. Notably, the overall prevalence of unmet needs showed a continuous decline over time. Interestingly, although the attrition rate for unmet health care needs decreased significantly during the COVID-19 pandemic, unmet dental care needs remained markedly higher than unmet medical needs. Therefore, it is essential to implement more targeted measures to address and prevent these gaps in dental care.

### Comparisons With Previous Studies

Numerous studies have investigated unmet health care and dental care needs during the COVID-19 pandemic [36]. These studies explored trends and related factors among Korean adults, such as low income [37], urban residence [21], unmarried status, and being female. Additionally, other research on the relationship between unmet medical needs and the COVID-19 pandemic focused on specific populations, including older adults [38], patients with chronic diseases, those with diabetes mellitus or poor glycemic control [39], community-dwelling older adults [40], and young adults [41]. Several studies also examined unmet medical needs in the general adult population in Austria (N=2000) [42], Canada (N=23,972) [43], and the United States (N=1,483,378) [44]. These studies found that older adults, inactive individuals, and retirees were particularly susceptible to experiencing unmet medical needs [42]. Conversely, other research studies indicated that younger individuals are more vulnerable to unmet medical care needs [43,45]. Identified risk factors also include having a chronic disease, being an immigrant [43], and being female [45]. However, most of these studies were limited to short-term analyses, small or specific target populations, and data from either before or during the pandemic. By contrast, our study offers comprehensive insights into risk factors and trends for unmet health care and dental care needs over the years 2009 to 2019 (before the pandemic) and 2021 to 2022 (during the pandemic) using a substantial data set of 2.7 million individuals.

### Plausible Mechanism

The prevalence of unmet health care and dental care needs may have decreased due to the economic relief provided by the medical insurance system, increased accessibility, and reduced waiting times resulting from the growth in the number of hospitals and clinics. However, the slower rate of decline in unmet needs could also be partially attributed to patients’ reluctance to visit hospitals or clinics due to concerns about potential infection [13,46]. Many experts emphasized that the decrease in hospital visits was not due to a reduction in the number of patients, but rather to anxiety, concerns about infection, and executive orders to close hospitals [4,13].

Significant demographic and social variables have been identified in individuals with a high prevalence of unmet health care needs. Notably, these individuals are often in their 40s, predominantly female, unmarried, with lower income brackets, and minimal levels of education. Blue-collar workers, in particular, are more likely to experience injuries during labor and see a more rapid decline in physical ability as they age. Female blue-collar workers, in particular, reportedly have higher rates of chronic diseases, nontreatment, and poor health behaviors [47]. Additionally, the high unmet health care needs among those with low income, especially basic livelihood security recipients, are largely attributed to the economic burden associated with medical services. Individuals with a low level of education often experience a high rate of unmet health care needs, which is associated with lower income, poor health conditions, and high smoking rates. Similarly, people aged 40-59 years with high working hours and those living in rural areas with relatively low access to medical care are significantly influenced by time burdens, which appear to contribute to unmet health care needs.

The relationship between lifestyle choices and unmet health care needs is evident. Smokers, individuals who rarely exercise, those with poor subjective health, and those who are underweight often experience unmet needs, despite receiving medical services, due to their higher use of health care. Additionally, poor mental health and low self-esteem, including depression, are known to adversely affect unmet medical needs [48]. Unmet health care needs are particularly high among unmarried individuals and women, who are more vulnerable to mental health problems [49]. While the risk factors for unmet dental needs are similar to those for unmet medical needs, unmet dental care needs are notably higher among patients with diabetes mellitus. This may be due to the particularly adverse effects of diabetes on oral health, leading to greater dental care needs [50].

In summary, unmet health care needs were found to be high in groups with significant medical demands and economic, time, and psychological burdens. The β_diff_ analysis revealed that, after the COVID-19 pandemic, individuals who are older, unemployed, have low income, poor subjective health, low weight, high blood pressure, diabetes, urban residents, and those who smoke or consume alcohol were particularly vulnerable. Groups with high health care demands, such as those with poor subjective health, low weight, high blood pressure, and diabetes, as well as those with weak social or economic foundations (older adults, unemployed, and low income), were more affected by COVID-19. Urban areas also experienced greater changes due to higher population densities and stricter infectious disease regulations [51]. The decrease in dental care needs during the pandemic is attributed to COVID-19’s respiratory transmission, which led to reluctance to visit dentists due to the need to remove masks.

### Limitations

This study has several limitations. First, unmet medical care needs were defined based on patients’ subjective judgments, which might lead to discrepancies with actual needs. Second, values such as health level and BMI were self-reported, although self-reported BMI is generally reliable. Third, data from 2010 and 2020 were excluded due to the absence of questions regarding unmet health care needs, which may affect trend analyses for the COVID-19 pandemic period. Finally, the study focused solely on Korean adults, which may limit the generalizability of the findings to global trends.

### Conclusions

This long-term, representative, population-based study shows that while the prevalence of unmet health care needs has generally declined, there was a noticeable decrease during the COVID-19 pandemic. Addressing this issue requires implementing more detailed measures to prevent the emergence of unmet medical and dental care needs.

## Abbreviations

KCHS: Korea Community Health Survey
PR: prevalence ratio

## Data Availability

Data are available on reasonable request. The study protocol and statistical code are available from DKY. The study data set is available from the Korean Disease Control and Prevention Agency through a data use agreement.
